# The Headache Psychologists’ Role in Pediatric and Adult Headache Care: A Qualitative Study of Expert Practitioners

**DOI:** 10.1007/s10880-023-09972-2

**Published:** 2023-10-15

**Authors:** Amy S. Grinberg, Teresa M. Damush, Hayley Lindsey, Laura Burrone, Sean Baird, Stanley Curtis Takagishi, Ivy Snyder, Roberta E. Goldman, Jason J. Sico, Elizabeth K. Seng

**Affiliations:** 1 VA Connecticut Healthcare System, West Haven, USA; 2 Headache Centers of Excellence Research and Evaluation Center, Veterans Health Administration, West Haven, USA; 3 Pain, Research, Informatics, Medical Comorbidities, and Education (PRIME) Center, VA Connecticut Healthcare System, West Haven, USA; 4 Richard L. Roudebush VA Medical Center, Indianapolis, USA; 5 Indiana University School of Medicine, Indianapolis, USA; 6 Regenstrief Institute, Inc, Indianapolis, USA; 7 James A Haley VA Hospital, Tampa, USA; 8 Ferkauf Graduate School of Psychology, Yeshiva University, Bronx, USA; 9 Warren Alpert Medical School of Brown University, Providence, USA; 10 Harvard T.H. Chan School of Public Health, Boston, USA; 11 Yale School of Medicine, New Haven, USA; 12 The Saul R. Korey Department of Neurology, Albert Einstein College of Medicine, Bronx, USA; 13 VA Connecticut Healthcare System Headache Center of Excellence, National Programs Center–Mailing Code 689GF VA Annex, 200 Edison Road, Orange, CT 06477 USA

**Keywords:** Headache, Psychology, Behavioral interventions, Qualitative methods

## Abstract

**Objective:**

We examined the perspectives of expert headache psychologists to inform best practices for integrating headache psychologists into the care of children and adults with headache disorders within medical settings.

**Background:**

Headache disorders are prevalent, chronic, and disabling neurological conditions. As clinical providers trained in evidence-based behavior change interventions with expertise in headache disorders, headache psychologists are uniquely positioned to provide behavioral headache treatment.

**Methods:**

In 2020, we conducted semi-structured interviews with a purposive sample of expert headache psychologists working across the United States. Open–ended questions focused on their roles, clinical flow, and treatment content. Interviews were audio-recorded, transcribed, de-identified, and analyzed using a rapid qualitative analysis method.

**Results:**

We interviewed seven expert headache psychologists who have worked for an average of 18 years in outpatient settings with pediatric (*n* = 4) and adult (*n* = 3) patients with headache. The themes that emerged across the clinical workflow related to key components of behavioral headache treatment, effective behavioral treatment referral practices, and barriers to patient engagement. The expert headache psychologists offered evidence-based behavioral headache interventions such as biofeedback, relaxation training, and cognitive behavioral therapy emphasizing lifestyle modification as standalone options or concurrently with pharmacological treatment and were of brief duration. Participants reported many of their patients appeared reluctant to seek behavioral treatment for headache. Participants believed referrals were most effective when the referring provider explained to the patient the rationale for behavioral treatment, treatment content, and positive impact on headache activity, functioning, and quality of life. Barriers cited by participants to integrating headache psychology into headache care included the paucity of psychologists with specialized headache training, lack of insurance reimbursement, limited patient time to seek behavioral treatment, and inadequate patient knowledge of what behavioral treatment entails.

**Conclusion:**

Headache psychologists are often core members of multidisciplinary headache teams offering short-term, evidence-based behavioral interventions, both as a standalone treatment or in conjunction with pharmacotherapy. However, barriers to care persist.

Enhancing referring providers’ familiarity with psychologists’ role in headache care may aid successful referrals for behavioral interventions for headache.

**Supplementary Information:**

The online version contains supplementary material available at 10.1007/s10880-023-09972-2.

Approximately three billion people live with migraine or tension-type headache globally (Stovner et al., 2018). Within the United States (US), 1.4% of children (Tumin et al., 2018) and 14% of adults (Burch et al., 2015) are living with chronic headache (Hommer et al., 2021), defined as 15 or more days per month with headache (Headache Classification Committee of the International Headache Society, 2018). More recently, the prevalence of headache disorders within children has increased (Nieswand et al., 2020). Headache disorders are common and disabling, burdening those living with headache and resulting in significant direct and indirect costs to society (Adams et al., 2015; Burch et al., 2018; Buse et al., 2012, 2016; Lipton et al., 2007; Serrano et al., 2013). Despite this, headache disorders are insufficiently diagnosed and treated worldwide (World Health Organization, 2011). Managing headache disorders can be complex, often requiring preventive and acute treatment options (Silberstein et al., 2012). Behavioral interventions, such as biofeedback, relaxation training, and cognitive behavioral therapy, have demonstrated evidence to reduce headache frequency and disability (Penzien et al., 2015) and can be incorporated across each step of care (Singer et al., 2015), ranging from provider-delivered interventions to specialty care referrals.

Headache psychologists represent essential members of multidisciplinary headache care teams. A headache psychologist is a licensed clinical psychologist who specializes in the assessment, treatment, and management of headache disorders. They have extensive training and expertise in psychological and behavioral aspects of pain/headache. They are uniquely positioned to provide behavioral treatments for pediatric and adult populations with headache by helping patients manage lifestyle factors such as stress, sleep, and physical activity, which impact headache symptomatology (Nicholson, 2010; Rosenberg et al., 2018). Headache psychologists can also aid patients with medication management difficulties (Nicholson, 2010) and address common psychiatric comorbidities. Implementing behavioral interventions within a stepped-care approach (Baskin & Smitherman, 2009) may also be advantageous, given the complex management of headache disorders and challenges commonly occurring with behavioral change efforts (Singer et al., 2015). Patients have described numerous barriers to initiating behavioral treatment (Minen et al., 2018). However, little is known about the perspective of expert headache psychologists regarding barriers to integrating psychologists on headache care teams and disseminating behavioral treatments to headache patients. Additionally, while there are emerging core competencies being established for those who specialize in pain psychology (Wandner et al., 2019) there is no formal definition or core competency requirements for headache psychologists who work with adults or children (Law, Palermo, & Walco, 2012), thus understanding the role headache psychologists play in headache care is invaluable.

This qualitative study sought to fill this gap by interviewing expert headache psychologists to understand their perspective on integrating psychologists into a headache practice and barriers to integration and dissemination of behavioral treatments to headache patients. These data will inform our understanding of the role psychologists can play in managing primary and secondary headache disorders within pediatric and adult populations, with a focus on understanding the roles psychologists have within the headache care team, clinical flow, and treatment content, which will enhance understanding of best practices.

## Methods

### Sample and Recruitment

Between June and September 2020, two study team members (ASG & EKS) identified potential participants by examining a list of psychologists who were members of the behavioral section of the American Headache Society and/or part of the Special Interest Group of the Society for Behavioral Medicine and had published articles related to headache within the past two years. Team members then contacted potential participants via email to invite them to participate. We selected a purposive sample (Crabtree & Miller, 1992) of expert headache psychologists. We contacted headache psychologists who were working within a healthcare system in the United States (US) (academic institutions/large hospital-based systems), actively involved in national headache organizations, such as the American Headache Society, and/or having published peer-reviewed articles in the field of headache medicine and health psychology within the last two years. We selected this purposive sample as we wanted to ensure that we captured the perspectives of expert clinicians who were knowledgeable about the latest evidence-based interventions and practices in headache care and who were therefore likely at the forefront of headache care within the US. We selected clinicians from various academic and clinical settings with different levels of experience in treating headache disorders across regions of the US. Regarding the level of activity required to meet this criterion, we looked for evidence that the providers were actively involved in national headache organizations, such as serving in leadership roles, attending annual meetings, or presenting at conferences.

We contacted 13 headache expert psychologists who specialize in headache disorders within clinical and research settings. We could not reach five, and one was lost to follow-up. We conducted interviews until we met data saturation and no new themes emerged.

The final sample of participants were located across the US, including the following regions: West, Southwest, Midwest, Northeast, and Southeast.

### Interview Guide Development and Data Collection

The study team comprised experts in headache medicine, headache psychology, medical anthropology, health services research, implementation science, and qualitative and mixed methods. The team developed an interview guide based on the literature and experience of the study team (see supplemental materials, Table 2). The guide included 44 core, open-ended questions supplemented by prompts that focused on psychologists’ roles, clinical flow, care delivery, treatment content, and patient outcome metrics.

Three study team members (ASG, REG, & TMD) with prior experience with qualitative interviewing were trained to use the interview guides and conducted semi-structured interviews by telephone. In addition, a research assistant (HL or LB) was also on the call and completed took running notes during the interview in order to complete a note template, which included key concepts that the research team identified, interviewer perceptions, and unexpected insights. Each interview lasted between 60 and 90 min. Team members continued collecting data until they reached data saturation, based on iterative data analyses and team discussions (Fusch & Ness, 2015; Saunders et al., 2018). Data saturation was defined as the point at which additional interviews were no longer providing new information related to the research questions (Saunders et al., 2018).

### Data Analysis

All interviews were audio-recorded, professionally transcribed, and de-identified. First, interviews were analyzed using a rapid analysis approach (Hamilton & Finley, 2019) that allowed us to quickly analyze and interpret the data from our interviews. Specifically, we used notes templates to create a topical matrix, which was then reviewed by two members of the research team. These two analysts compared findings across interviews to identifies common themes and patterns. Next our team of four analysts reviewed the matrix, identified content, including patterns and themes. These four analysts worked together to ensure that any discrepancies were resolved and that the final interpretation was robust and accurate. The primary author then examined all the data to finalize themes and interpretation of the data. Our analysis approach followed best practices for qualitative research and allowed us to accurately identify and interpret themes and patterns in our data (Tong et al., 2007).

### Ethical Considerations

The Veterans affairs connecticut healthcare system research and development and institutional review board committee, west haven, connecticut approved this research study. Before starting each interview, the participant provided verbal consent to participate in an audio-recorded interview.

## Results

We interviewed seven expert headache psychologists (four men and three women) who delivered care for an average of 18 years (range = 2–40) in outpatient settings to pediatric (*N* = 4) and adult (*N* = 3) patients with headache. Following are our findings grouped into three themes that emerged from data analysis.

### Headache Psychologists Tailor Treatment Plans to Patient Preferences and Symptom Presentation While Incorporating Various Headache-Specific Behavioral Techniques

To aid in tailoring treatment to patient preference and symptom presentation, all the headache psychologists interviewed conducted an initial comprehensive biopsychosocial assessment to understand pertinent information related to the patient’s medical history, headache patterns, and psychiatric comorbidities.

Headache psychologists in this study provided a range of evidence-based behavioral interventions, most commonly biofeedback, cognitive behavioral therapy, relaxation training, and lifestyle modification (for stress, sleep, exercise, and diet). All expert headache psychologists provided brief interventions (typically six to eight appointments) tailored to patients’ preferences and their unique symptom presentation, “There’s a very nice body of work describing efficacy for biofeedback, relaxation therapy, and Cognitive Behavioral Therapy.” ^[PSY_01]^ All interviewees explained that education is a core part of treatment and is often successfully incorporated even within the initial assessment period, “It would be helpful for them to build skills that will help their headache and that they understand the biopsychosocial model of the mind–body connection.”.^[PSY_05]^

There were some differences between headache psychologists who work with pediatric populations and those who work with adults. Pediatric headache psychologists tended to involve family members more extensively at various stages of treatment to aid treatment initiation and maintenance, “The family component of making sure that the people that are involved in the person’s life are making modifications and supporting them is a foundational treatment component.” ^[PSY_05]^ Additionally, they routinely modified their treatment techniques based upon the child’s age and developmental level.

“The younger the patient, the more basic the relaxation, the better. Just based on development. Deep breathing is very effective with little kids, and parents can coach deep breathing. Progressive muscle relaxation is pretty effective in little kids too because it’s very tangible. You do it. You feel it. You see it. Imagery is a little bit more likely to be more adolescence and mid adolescence to late adolescence to be effective.” ^[PSY_02]^

Expert headache psychologists considered biofeedback, cognitive behavioral therapy, and relaxation strategies core behavioral interventions for headache. In addition, they typically incorporated several other techniques, including motivational interviewing, and discussed the potential benefits of lifestyle modification (sleep hygiene, exercise, nutrition, and stress management) on headache symptomatology. Finally, they highlighted the importance of close collaboration with referring providers, such that referring providers provide recommendations for both medication and lifestyle management, then psychologists can utilize techniques to reinforce and implement those recommendations in the daily lives of people with headache.

“Lifestyle components that have been scientifically shown to matter in migraine management, sleep hygiene, stress management, exercise, diet and nutrition. Those are areas that are very important in migraine management. That’s education that the physician probably will do as well, and the psychologist not only would reinforce what the physician said but the psychologist might help pave the way to success by using motivational interviewing, by using CBT strategies, to actually help the patient implement these recommendations from their physician.” ^[PSY_01]^

Headache psychologists frequently used acceptance and commitment therapy and other mindfulness-based treatments to improve the quality of life of both pediatric and adult populations, “Some of our psychologists will expand from basic relaxation and cognitive behavioral packages to mindfulness, and even acceptance and commitment therapy.”.^[PSY_02]^

Given the unique and complex nature of headache disorders, headache psychologists commented that formal education and prior supervised clinical training utilizing cognitive behavioral therapy techniques are necessary to work with this population. Therefore, they suggested having at least a Master’s degree level of training in cognitive behavioral therapy is needed, and a Ph.D./Psy.D. is preferred. Moreover, they stressed that experience working specifically with patients with headache disorders is crucial to identifying when it is appropriate to refer back to a neurologist as red flag symptoms present, “Formal education and supervised clinical training utilizing cognitive behavioral therapy with this population is important.” ^[PSY_07]^ “A Ph.D. is preferred but an MS-level clinician who has comfort with cognitive behavioral therapy could do this just fine.”.^[PSY_01]^

### Fostering Strong Relationships Between Providers And Patients While Communicating the Rationale For Behavioral Interventions Results in a More Effective Referral

Headache psychologists most commonly received referrals from their neurology, nursing, physiatry, obstetrics and gynecology colleagues. Most interviewees discussed the benefits of multidisciplinary headache teams, which allowed for regular communication and greater collaboration between all disciplines, resulting in a more streamlined and effective referral process, “Every new patient that comes to the interdisciplinary clinic sees a neurologist, the nurses, the psychologists …and as a team we come up with what we think the best treatment plan is.”.^[PSY_02]^

Interviewees noted that multidisciplinary treatment teams reinforced the importance and value of a multimodal approach to headache care, increasing patient confidence in the behavioral treatment. While not always feasible, interviewees noted the best collaborative care includes continued coordination between the medical providers and psychologists through warm-handoffs to help strengthen the patient-provider relationship, “I think that really requires a great team approach.”.^[PSY_04]^

**Table 1:** Best practice recommendations described by expert headache psychologists

Theme	Recommendation
Tailoring treatment plans to patient preferences and symptom presentation while incorporating various headache-specific behavioral techniques	Headache psychologists may find it beneficial to conduct a comprehensive biopsychosocial assessment during their initial intake appointment
	Headache psychologists provide psychoeducation and may offer a range of tailored, short-term, headache-specific, evidence-based behavioral interventions (biofeedback, cognitive-behavioral therapy, relaxation training, lifestyle modification, acceptance and commitment therapy, mindfulness-based interventions)
	Providers offering behavioral interventions, such as cognitive behavioral therapy and biofeedback, should receive formal training in each modality they provide
	Headache psychologist who work with pediatric populations may wish to involve family members at various stages of treatment and modify their treatment techniques based on the child’s age and developmental level
Fostering strong relationships between providers and patients while communicating the rationale for behavioral interventions results in a more effective referral	Given the high volume of patients that seek headache care in primary care settings, it may be beneficial for headache medicine to utilize models already in place in primary care departments to integrate psychologists into routine headache practice
	Behavioral treatments for headache are most effective for patients presenting with headache as primary concern. Providers may wish to consider referring to specialty mental health services for patients whose primary concern is a mental health diagnosis
	Referring clinicians may benefit from additional education and training on what behavioral treatment entails, which may aid effective referrals. They may provide initial recommendations about life style management and headache psychologists can then utilize techniques to reinforces and implement recommended changes
	Incorporating headache psychologists into multidisciplinary teams allows for greater communication and collaboration between providers, which can help foster strong relationships between providers and their patients
Increasing the availability of headache psychologists, addressing headache-related stigma, and advocating for improved treatment cost reimbursement may address barriers to patient engagement in behavioral headache interventions	Shoring up workforce development initiatives through increased graduate-level training in headache psychology and continuing education focused on headache for behavioral treatment providers who meet the minimum qualifications (e.g., a full course of formal training in the treatment modality, such as biofeedback or cognitive behavioral therapy) may address the paucity of headache trained psychologists in the US
	Referring providers may find it beneficial to be cognizant of the language they use when making a referral to a headache psychologist in order to address misconceptions, biases, and stigma associated with behavioral interventions for headache
	Continue advocacy efforts to address barriers to engagement, such as high out of pocket costs of behavioral treatment

Headache Psychologists interviewed noted that traditional psychiatric and mental health services might be more appropriate for patients whose primary presenting concern is psychiatric. In contrast, headache psychologists may be best situated to treat patients whose primary presenting concern is headache. Most psychologists interviewed reported that many of their patients did not fully understand why their providers referred them for behavioral interventions for headache. Consequently, misconceptions about the rationale for referral (e.g., patients believing the referral to a headache psychologist was primarily made to address psychiatric, rather than headache, presenting concerns) resulted in patients declining or not following up on the referral. Therefore, interviewees recommended providing medical clinicians with additional education and training on what behavioral treatment entails to aid effective referrals. When referring providers have treatment buy-in and a clear understanding of the rationale, expectations, and impact of treatment on disease activity, functioning, and quality of life, they can more effectively communicate this to their patients in a clear and relevant way before placing a referral.

“The most important factor is how the neurologist talks about the referral. If they have [treatment] explained in a way that feels palatable and hopeful then they have a lot of buy-in and they follow up with the appointment, versus if they don’t understand what treatment is and they feel like they’re just being referred to therapy because their provider thinks that there’s something wrong with them or they’re crazy.” ^[PSY_05]^

As one psychologist asserted, “The handoff piece is very important. I think how the provider frames the experience, what the patient is going to experience, what they can expect in terms of benefit. Make it seem as valuable as medication or medical approaches.”.^[PSY_01]^

### Increasing the Availability of Headache Psychologists, Addressing Headache-Related Stigma, and Advocating for Improved Treatment Cost Reimbursement May Address Barriers to Patient Engagement in Behavioral Headache Interventions

All headache psychologists interviewed encountered several barriers when engaging patients in behavioral interventions. Interviewees found patients are often reluctant to engage in behavioral treatment due to misconceptions, bias, and stigma related to behavioral treatment. Headache psychologists participating in this study stated their patients thought their providers referred them because they did not believe their pain and symptoms were real or because they had psychiatric symptoms needing treatment. The interviewed psychologists asserted that providers often presented behavioral management of headache as a last resort for those patients who are refractory to medical treatment options rather than as a core component of multidisciplinary headache treatment plans, “Language is often focused on try the medicine first, and then if you don’t succeed with the medication, then we will add psychology. It’s always viewed as a last hope for the patient, and I think that that kind of sours the experience.”.^[PSY_08]^

Another factor that resulted in barriers to patient engagement is the paucity of headache-trained psychologists in the US. Interviewees noted that many patients struggled to find a health psychologist who specializes in behavioral headache interventions and has availability. One headache psychologist noted s/he was the only psychologist on staff in their headache center, “There are not enough of us [psychologists], and not enough of us have been trained in evidence-based care. There are not enough behavioral trained people to even come close to treating them.” ^[PSY_02]^ Moreover, patient engagement can be challenging because of out-of-pocket costs of treatment, having little or no insurance coverage for treatment expenses, and difficulties devoting time to seeking in-person care due to conflicting responsibilities such as employment, childcare, and attending other medical appointments. Interviewees concluded that patients who lived near facilities that provided behavioral headache treatment and had the means to pay are the most likely to receive and engage in treatment.

“It can be very hard to find a provider, let alone accepting any insurance, both peds and adults. That seems to be very big barriers, as well as time and accessibility, getting off work, getting childcare, driving in traffic to wherever it is, hours, and then someone with migraine may not feel well.” ^[PSY_01]^

***COVID-19:*** These interviews were conducted during the height of the COVID-19 pandemic, which had a significant impact on healthcare delivery, and may have influenced patient and provider experiences. Of the six questions that were asked pertaining to the impact of the pandemic on healthcare delivery, providers only commented that they had to switch to virtual care delivery and were unable to provide biofeedback as a treatment modality.

## Discussion

This qualitative study elucidated the perspective of expert headache psychologists regarding best practices for integrating psychologists in the routine care of headache patients, barriers to integrating psychologists at an administrative level, and uptake of behavioral treatments at a patient level in both pediatric and adult populations. Psychologists interviewed described best practices at multiple levels. Administrative best practices included psychology being integrated into a multidisciplinary headache care team. Referral best practices included referring patients for whom headache is their primary presenting concern and describing behavioral treatment clearly to the patient as a method to manage headache (rather than primarily treating psychiatric illness). Finally, behavioral treatment best practices included providing headache-specific, evidence-based care tailored to the patient’s preferences, presenting concern and symptom presentation, and behavioral assessment.

In this study, psychologists described stigma (Golberstein, Eisenberg, & Gollust, 2008; Sickel et al., 2014) associated with referral to a psychologist as a barrier to patient engagement with treatment. Our findings highlight the importance of referring providers in validating their patients’ experiences. Psychologists most commonly provide mental health treatment; it is reasonable that, without any alternative explanation, patients interpret a referral to a psychologist as the provider considering their headache disease to be “in your head,” and primarily psychological. Interviewees asserted referring providers should be familiar with the rationale for behavioral interventions to reduce headache symptoms and emphasize to patients that the referral is specifically for headache care. It may be beneficial to conceptualize behavioral interventions as a core component of headache treatment (Singer et al., 2015) rather than as a last resort for patients who are treatment-refractory or present with psychiatric concerns. Headache psychologists could support providers with training about how and when to “make the pitch” for behavioral interventions.

Based on our study findings, evidence-based interventions for headache most commonly used by expert headache psychologists include biofeedback, cognitive behavioral therapy, relaxation training, and, more recently, mindfulness-based approaches, such as acceptance and commitment therapy (ACT). Behavior change is challenging, and brief education rarely engenders long-lasting change (Arlinghaus & Johnston, 2017). Headache psychologists can apply their training in behavior change methodologies across the care spectrum, including in creating patient education material to be provided to all patients.

The headache psychology experts we interviewed strongly endorsed the importance of formal training in these behavioral interventions, specifically focusing on headache. However, the current training pipeline to produce headache psychologists is severely inadequate to handle the volume of patients who could benefit from the services. Shoring up workforce development initiatives through increased graduate-level training in headache psychology and continuing education focused on headache for behavioral treatment providers who meet the minimum qualifications (e.g., a full course of formal training in the treatment modality, such as biofeedback or cognitive behavioral therapy) may begin to fill that gap. Another potential approach to address the shortage of psychologists with specialized expertise in headache assessment and treatment is to focus on integrating existing psychological services into multidisciplinary headache clinics. This could involve training generalist psychologists to provide evidence-based headache treatments and working collaboratively with neurologists and other healthcare providers to provide comprehensive care to patients. Additionally, telehealth and other remote delivery options could be explored to increase access to headache psychology services in underserved areas. A more comprehensive and sustainable approach to addressing the shortage of headache psychologists may involve a combination of increased training and education, integration of existing psychological services into headache clinics, and use of technology-assisted treatments to improve access to care.

Notably, none of our interviewees described primary care as an important pipeline for headache referrals. Primary care initially treats the majority of people with migraine and tension-type headache (Becker et al., 2015), and psychologists are often integrated into primary care settings to provide brief disease-focused interventions (Baird et al., 2014; Horevitz & Manoleas, 2013; E. F., Palermo, T. M., & Walco, G. A. 2012; Saint-Pierre et al., 2018). Future research should evaluate the integration of headache psychologists in primary care settings.

Our findings align with the literature on the superiority of integrated, multidisciplinary care over co-located or referral-based care (Goldman et al., 2022; Singer et al., 2015). Barriers to accessing headache psychologists described by our study participants were consistent with obstacles outlined in the literature to integrating behavioral and physical health care in other settings (Druss & Newcomer, 2007). Headache medicine can benefit from utilizing models already in place in anesthesiology and primary care departments to integrate psychologists into routine headache practice, which may ultimately help with effective referrals to headache psychologists and address barriers to patient engagement. As headache medicine continues to mature as a field, the constitution of the multidisciplinary care team in headache centers will become increasingly important (Gaul et al., 2016; Steiner et al., 2019).

When considering differences between pediatric and adult practices, we found that psychologists who work with pediatric populations often find it helpful to incorporate family members within the treatment process. In addition, understanding family members’ role in supporting adult patients undergoing behavioral headache treatment may be helpful, given that lack of social support can result in increased stress and poorer self-rated health for people with headache (Westergaard, Lau, Allesøe, Andreasen, & Jensen, 2021).

Limitations of this study include that we interviewed psychologists who work in healthcare systems that rely on insurance reimbursement. It is possible that those who practice in managed care settings may differ concerning their identified barriers to patient engagement. Future research examining barriers to patient engagement and effective referral practices across various healthcare system structures may be beneficial. While we collected data from expert headache psychologists who practice in different regions in the US, multidisciplinary clinics can vary greatly regionally. They are frequently located in specialty/tertiary care centers where patients have already made it through the medical system to get to that stage. This may lead to differences in barriers and needs among that population, which should be explored in future research. Additionally, while leveraging expert headache psychologists in this study provides valuable insights into best practices, the results may not fully encompass the practices of a broader range of providers Future research focusing on non-expert headache psychologists, referring providers’, and patients’ perspectives may add to our current findings. Finally, as is typical of qualitative studies, we had a small sample size. However, we utilized a purposive sampling strategy of expert headache psychologists to enhance study credibility.

## Conclusion

Headache psychologists can serve as core members of multidisciplinary headache teams offering short-term, evidence-based behavioral interventions, such as biofeedback, cognitive behavioral therapy, and mindfulness-based approaches. However, there is a shortage of psychologists in the U.S. who specialize in headache, and high numbers of patients who could benefit from their services. The most effective referrals occur when referring providers explain to patients the treatment rationale, expectations, and anticipated positive impact of behavioral interventions on their headache disorder, functioning, and quality of life. Incorporating headache-specific training early within graduate school, and routine incorporation of psychologists in multidisciplinary headache centers, can undergird important initiatives to increase access to headache psychology care. Addressing barriers to treatment engagement and advocating for reimbursement of behavioral treatment for headache is necessary to improve access to care (Table 1).

### Supplementary Information

Below is the link to the electronic supplementary material.

Supplementary file1 (DOCX 17 KB)

## Data Availability

Not applicable.

## Abbreviations

(VHA): Veterans health administration
*(HCoE)*: Headache center of excellence
