# Supplementary material for: The Headache Psychologists’ Role in Pediatric and Adult Headache Care: A Qualitative Study of Expert Practitioners
Source: J Clin Psychol Med Settings. 2023 Oct 15;31(2):359–67. doi: 10.1007/s10880-023-09972-2 (PMC11102355; doi:10.1007/s10880-023-09972-2)
Supplement: Supplementary file 1 — Supplementary file1 (DOCX 17 KB) [file 10880_2023_9972_MOESM1_ESM.docx]

Supplemental Material

Table 2. Participant Interview Guide

| Interview Guide – Questions Asked |
| --- |
| 1. Please tell us about your role in providing care for patients with headache disorders. 2. How many years in total have you been providing headache care to patients? How long have you been working within your current center? In what department are you located? 3. What types of headache diagnoses do you see within your center? 4. What approximate percentage of patients do you see with various headache disorders (e.g. migraine, tension-type, cluster, post-traumatic headache)? 5. What are the age ranges and demographics of your patients? 6. What role do psychologists play in the management of patients with headache disorders in your healthcare organization? 7. How many other psychologists are there in your facility? Are you the only health psychologist in your headache clinical program? If no, how many others? 8. Does your clinic have a multidisciplinary approach to headache management (i.e. physician, advanced care provider, residents/fellows, pain psychologist, physical therapist, psychiatrist, etc.)? 9. In what settings do you see patients with headache disorders (inpatient, outpatient, intensive outpatient programs)? 10. Has the COVID pandemic modified the setting in which you see patients with headache disorders? 11. What types of treatment modalities do you use (individual, group, family therapy)? 12. How has the recent COVID19 pandemic changed or modified what treatment modalities you provide? 13. What types of psychological treatments are offered for patients with headache disorders within your center? 14. What specific techniques do you incorporate into your treatment? Do you incorporate any other therapies or techniques? Any changes since the COVID19 pandemic? 15. How effective do you think your patients perceive psychological treatments to be for relief of their headache symptoms? Which modalities are you thinking about as you answer this? 16. Which modalities do patients seem to stick with longer? 17. What do you think helps a patient initiate psychological treatment for their headache disorder? 18. What do you think hinders patients’ engagement in psychological treatments for their headache disorder? Why? 19. What barriers do you face in making psychological treatments available to your patients with headache disorders? How have you overcome these barriers? Has the COVID19 Pandemic introduced any additional barriers? 20. What recommendations can you share regarding how to implement psychological treatments for patients with headache disorders? Do you have any recommendations regarding implementing psychological treatments for Veterans specifically? 21. The VA has invested in complimentary integrative health during the past decade in response to Veterans preferences for alternative treatments. What are your thoughts about the role of Complementary and Integrative Health (CIH) in headache management? 22. How are patients referred to you for psychological treatment for their headache disorder? Typically, by whom? What typically triggers a referral? 23. What do you see as the core components of an effective referral? 24. When sharing information occurs with the referring provider, what types of communication modalities do you typically use? What type of information do you share with referring providers? On average, how often do you typically communicate with the referring provider? 25. Please describe what happens for a patient at their first appointment for psychological treatment? What are core components of the intake? Does your clinic use a headache intake form? If so, do patients complete it ahead of their visit or while they are in the waiting room? Are there any changes to this process during the COVID19 pandemic? 26. How many sessions do you typically see patients with headache disorders? 27. What is the typical length of each of your appointments? 28. What assessment measures or metrics do you routinely collect to monitor a patient’s response to treatment? How often do you administer each measure? 29. Do patients receiving care in your clinic use headache diaries? If so, in what format (e.g., paper, electronic)? How is information collected via a headache diary used by you and by your patients? How long do you ask your patients to complete their headache diary? What items are included in your headache diary? What have you found to be most useful to ask in the context of behavioral treatment? 30. To what extent do you make use of patient handouts? 31. In your professional opinion, what are useful patient resources to help manage headache disorders? [phone apps, devices, etc.]. 32. What treatment components do you think are foundational within psychological treatments for people with headache disorders? What do you think is missing from current treatment protocols? 33. When thinking about the psychological or behavioral headache care you provide, what do you think makes the biggest impact in the treatment for your patients with headache? 34. What has the least impact on your patients with behavioral headache treatment in your current healthcare system? 35. What are the main concerns of psychologists in terms of providing treatment to patients with headache today? 36. What are the main concerns of your patients with headache today? 37. Do you provide psychological treatment by telehealth services? If yes, for how long? How effective do you think the delivered treatment is? Why? What types of treatment are delivered via telehealth? 38. How difficult has it been to deliver treatment by telehealth? Why? What barriers have you encountered? How have your barriers/challenges been resolved? 39. What type of technology is used for telehealth? Provider/patient. Any technology problems? 40. How do you maintain treatment fidelity and quality through telehealth? 41. How do you promote patient adherence and minimize attrition and no shows? 42. Has your delivery of care been changed or modified due to the COVID19 pandemic? 43. What recommendations do you suggest for a clinical headache program setting up a new CBT telehealth program? 44. What other advice can you offer related to the implementation of behavioral and psychological treatments for patients with headache? |
